# Cortico-Subthalamic Field Potentials Support Classification of the Natural Gait Cycle in Parkinson’s Disease and Reveal Individualized Spectral Signatures

**DOI:** 10.1523/ENEURO.0325-22.2022

**Published:** 2022-11-10

**Authors:** Kenneth H. Louie, Ro’ee Gilron, Maria S. Yaroshinsky, Melanie A. Morrison, Julia Choi, Coralie de Hemptinne, Simon Little, Philip A. Starr, Doris D. Wang

**Affiliations:** 1 Department of Neurological Surgery, University of California, San Francisco, San Francisco, California 94143; 2 Department of Radiology, University of California, San Francisco, San Francisco, California 94143; 3 Department of Applied Physiology and Kinesiology, University of Florida, Gainesville, Florida 32611; 4 Department of Neurology, University of Florida, Gainesville, Florida 32608; 5 Department of Neurology, University of California, San Francisco, San Francisco, California 94143

**Keywords:** basal ganglia, deep brain stimulation, gait, Parkinson’s disease, sensorimotor cortex

## Abstract

The ability of humans to coordinate stereotyped, alternating movements between the two legs during bipedal walking is a complex motor behavior that requires precisely timed activities across multiple nodes of the supraspinal network. Understanding of the neural network dynamics that underlie natural walking in humans is limited. We investigated cortical and subthalamic neural activities during overground walking and evaluated spectral biomarkers to decode the gait cycle in three patients with Parkinson’s disease without gait disturbances. Patients were implanted with chronic bilateral deep brain stimulation (DBS) leads in the subthalamic nucleus (STN) and electrocorticography paddles overlaying the primary motor and somatosensory cortices. Local field potentials were recorded from these areas while the participants performed overground walking and synchronized to external gait kinematic sensors. We found that the STN displays increased low-frequency (4–12 Hz) spectral power during the period before contralateral leg swing. Furthermore, STN shows increased theta frequency (4–8 Hz) coherence with the primary motor through the initiation and early phase of contralateral leg swing. Additional analysis revealed that each patient had specific frequency bands that could detect a significant difference between left and right initial leg swing. Our findings indicate that there are alternating spectral changes between the two hemispheres in accordance with the gait cycle. In addition, we identified patient-specific, gait-related biomarkers in both the STN and cortical areas at discrete frequency bands that may be used to drive adaptive DBS to improve gait dysfunction in patients with Parkinson’s disease.

## Significance Statement

By recording from chronically implanted electrodes in the subthalamic nucleus and sensorimotor cortex in patients with Parkinson’s disease, we found power modulations across multiple frequency bands (4–30 Hz) during specific phases of the gait cycle. The coherence between subthalamic and cortical areas of each brain hemisphere also increases before contralateral leg swing. The data support the hypothesis that the basal ganglia and cortex coordinate alternating power and coherence fluctuations between hemispheres, which may indicate a mechanism to regulate continuous bipedal locomotion in humans. Last, we show that these putative biomarkers for gait can decode left and right gait events, implicating a potential use to drive future adaptive DBS algorithms.

## Introduction

Human walking is a complex motor task that requires the flexible coordination of reciprocal left and right leg movements. Natural upright walking consists of each leg alternating between the stance phase, when the foot is in contact with the ground, and the swing phase, when the foot is in the air; these two phases make up the “gait cycle,” comprised of a series of stereotyped events such as left and right heel-strikes and toe-offs.

The subthalamic nucleus (STN) and primary motor cortex are likely key nodes of the supraspinal network that regulates human gait, given the projection of the STN to the locomotor regions in the brainstem (Takakusaki, 2017) and its direct connections to the motor cortex via the hyperdirect pathway (Nambu et al., 2002). Understanding of the cortico-subthalamic network activities that underlie natural walking in humans is, however, limited because of methodological constraints. Scalp electroencephalography (EEG) studies have shown that natural overground walking is associated with fluctuations in the alpha (8–12 Hz), beta (13–30 Hz), and gamma (70–90 Hz) frequency ranges from the sensorimotor regions of healthy subjects (Gwin et al., 2011; Wagner et al., 2012; Seeber et al., 2015). Although EEG lacks the spatial resolution to discern whether these rhythms originate from the motor cortex or represent sensory feedback during walking, and are prone to movement artifacts. Basal ganglia field potentials recorded from implanted deep brain stimulation (DBS) leads of patients with Parkinson’s disease (PD) have also revealed modulation of beta oscillations (13–30 Hz) from the STN while stepping in place (Fischer et al., 2018; Hell et al., 2018; Tan et al., 2018) and during overground walking throughout the gait cycle (Arnulfo et al., 2018; Hell et al., 2018; Canessa et al., 2020). However, because aberrant beta oscillatory synchrony in the STN is a hallmark of akinesia in PD (Hammond et al., 2007; Little and Brown, 2014), and beta oscillations decrease with movement planning and execution in general, including those of the upper extremity (Kühn et al., 2004; Wingeier et al., 2006; Eisinger et al., 2020), whether these subthalamic beta modulations represent a biomarker of specific gait events is unclear. Finally, little is known about cortical–subthalamic interactions during the natural gait cycle.

Our hypothesis is that the STN interacts with the motor cortex in a temporal-specific manner to coordinate reciprocal leg movements to generate effective bipedal locomotion. We investigated the cortical–subthalamic circuit dynamics of natural walking from three patients with PD without major gait disturbances in the on-mediation state to capture the most physiological gait possible. Patients were implanted with chronic bilateral STN DBS leads and sensorimotor cortex electrocorticography paddles. Neural oscillatory activities were simultaneously and wirelessly streamed from the bilateral primary motor cortex (M1) and somatosensory cortex (S1) as well as the STN during overground walking, and were synchronized to external gait kinematic sensors. Our aims were as follows: (1) to characterize the oscillatory signatures of natural walking from the STN and sensorimotor cortices; (2) to identify cortico–subthalamic circuit coherence changes throughout the gait cycle; and (3) to determine the accuracy of gait event decoding (i.e., heel-strike or toe-off) based on these cortical and subthalamic oscillatory signatures.

## Materials and Methods

### Subjects and electrode reconstruction

Three male subjects with idiopathic PD undergoing evaluation for DBS surgery were enrolled at the University of California, San Francisco. Subjects did not exhibit major gait impairments, with Movement Disorder Society Unified Parkinson's Disease Rating Scale III (UPDRS III) postural instability and gait subscores on medication between 1 (slight) to 2 (mild; Table 1). All subjects provided written informed consent (ClinicalTrials.gov ID: NCT-03582891).

**Table 1:** Subject demographics

ID	Age/sex	Disease duration	DBS target	UPDRS III total off-meds	UPDRS III total on-meds	UPDRS III PIGD* on-meds
Subject 1	42/M	06	STN	41	14	2
Subject 2	58/M	09	STN	34	09	1
Subject 3	61/M	05	STN	35	12	1

M, Male.

**Figure 1.**
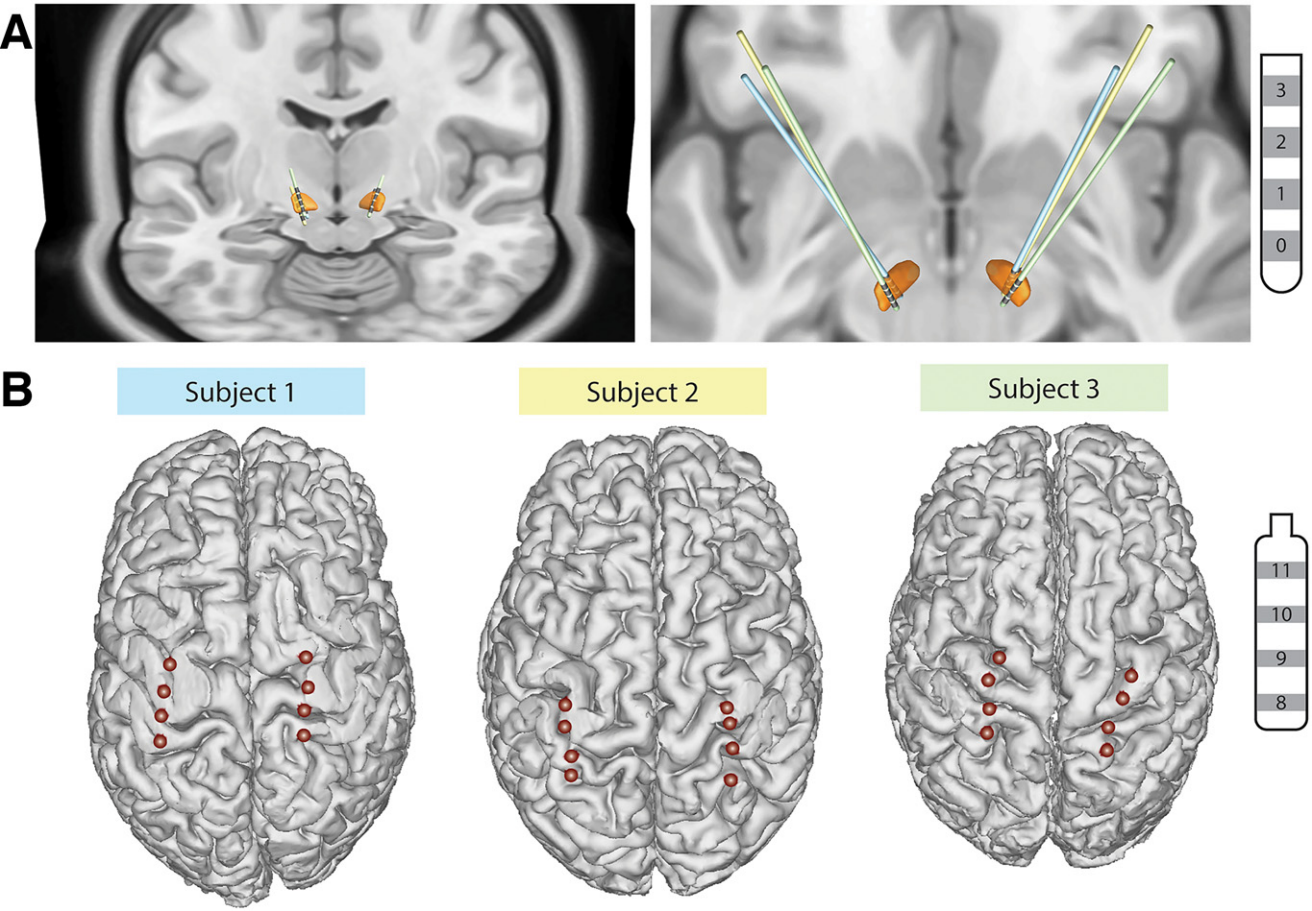
DBS and cortical lead localization. ***A***, 3D reconstructions of all DBS lead locations in the STN (orange). Individual subject’s leads are shown in by different colors. ***B***, 3D reconstructions of cortical electrode paddle location. The two most anterior contacts overlie the M1, while the two most posterior contacts overlie the S1.

All subjects underwent bilateral implantation of quadripolar DBS leads into the STN (model 3389, Medtronic), quadripolar cortical paddle overlying the sensorimotor cortices (model 0913025, Medtronic), connected to bilateral investigational sensing pulse generators (Summit RC+S model B35300R, Medtronic) as previously described (Fig. 1; Gilron et al., 2021a). Each RC+S device was connected to an STN DBS electrode and a cortical paddle from the same brain hemisphere.

DBS and cortical electrode localization were performed using 2-month postoperative computed tomography (CT) images fused with preoperative T1-weighted MRI images. STN DBS lead reconstruction was performed using the DISTAL atlas and TRAC/CORE algorithm available within LEAD-DBS, an open-source MATLAB toolbox (Horn and Kühn, 2015; Ewert et al., 2018). Intracranial EEG Anatomical Processing and Electrode Reconstruction Pipeline (https://edden-gerber.github.io/ecog_recon/) was used for cortical paddle reconstruction. T1 images were parcellated and converted into a standardized cortical surface mesh using FreeSurfer (Dale et al., 1999) and AFNI SUMA (Saad et al., 2004). Cortical contacts were then manually identified on the CT images in BioImage Suite (Section of Bioimaging Sciences, Department of Diagnostic Radiology, Yale School of Medicine; http://www.bioimagesuite.org), and the electrode coordinates were projected onto the standardized mesh using a gradient descent algorithm in MATLAB.

### Neural recordings and gait kinematic measurements during natural walking

Subjects walked overground at their preferred speed for 2 min in a straight path of at least 15 feet before turning around. All subjects were receiving their typical dose of parkinsonian medication during the task. In all subjects, local field potentials (LFPs) were recorded from the following two STN electrode pairs: ventral STN (contacts 2 and 0) and dorsal STN (contacts 3 and 1), where contact 0 is in the ventral STN, contact 3 is just above the dorsal border, and contacts 1 and 2 are in the motor territory based on microelectrode mapping (Fig. 1*A*). The two cortical electrodes recording configuration were contacts 9 and 8 (S1) and contacts 10 and 11 (M1), based on an imaging reconstruction. LFPs were sampled at 500 Hz and passed through a preamplifier high-pass filter of 0.85 Hz and a low-pass filter of 450 Hz. Accelerometry data from the Summit RC+S system was sampled at 64 Hz. All data from the RC+S system were extracted and analyzed using open-source code (https://github.com/openmind-consortium/Analysis-rcs-data).

Gait kinematic data were collected using two wireless sensor systems: Trigno system (Delsys) and MVN Analyze (Xsens). The Delsys sensors included two Avanti force-sensitive resistor (FSR) adapters, two Avanti goniometer adapters, and two Trigno surface electromyography (EMG) sensors with a built-in accelerometer. The Avanti adapters were placed bilaterally on the shank of the leg, and the EMG sensors were placed on top of both RC+S and used for synchronization (see below). Each FSR adapter was attached to four FSRs (model DC:F01, Delsys) placed under the calcaneus, hallux, first metatarsal (1MT), and fifth metatarsal (5MT). A digital goniometer (SG110/A) was placed next to the lateral malleolus. The Xsens system is composed of 14 inertial measurement unit sensors placed over the entire body and limbs for wireless motion tracking.

### Data analysis

LFP and gait kinematic data were synchronized by aligning the acceleration peaks captured by the RC+S Trigno sensors (Delsys) over the RC+S and Xsens accelerometry. Four signal-processing methods were applied to the LFP signals using built-in MATLAB functions, as follows: continuous wavelet transform (CWT; “cwt” function), wavelet coherence (“wcoherence” function), short-time Fourier transform (“spectrogram” function), and power spectral density (PSD; “spectrogram” function with 1 s window, 90% window overlap, and a transform length of 512 data points). We used wavelet transformation because it has greater low-frequency resolution. We also used the Fourier transform because this is the on-board spectral decomposition method used by the RC+S system (Sellers et al., 2021).

**Figure 2.**
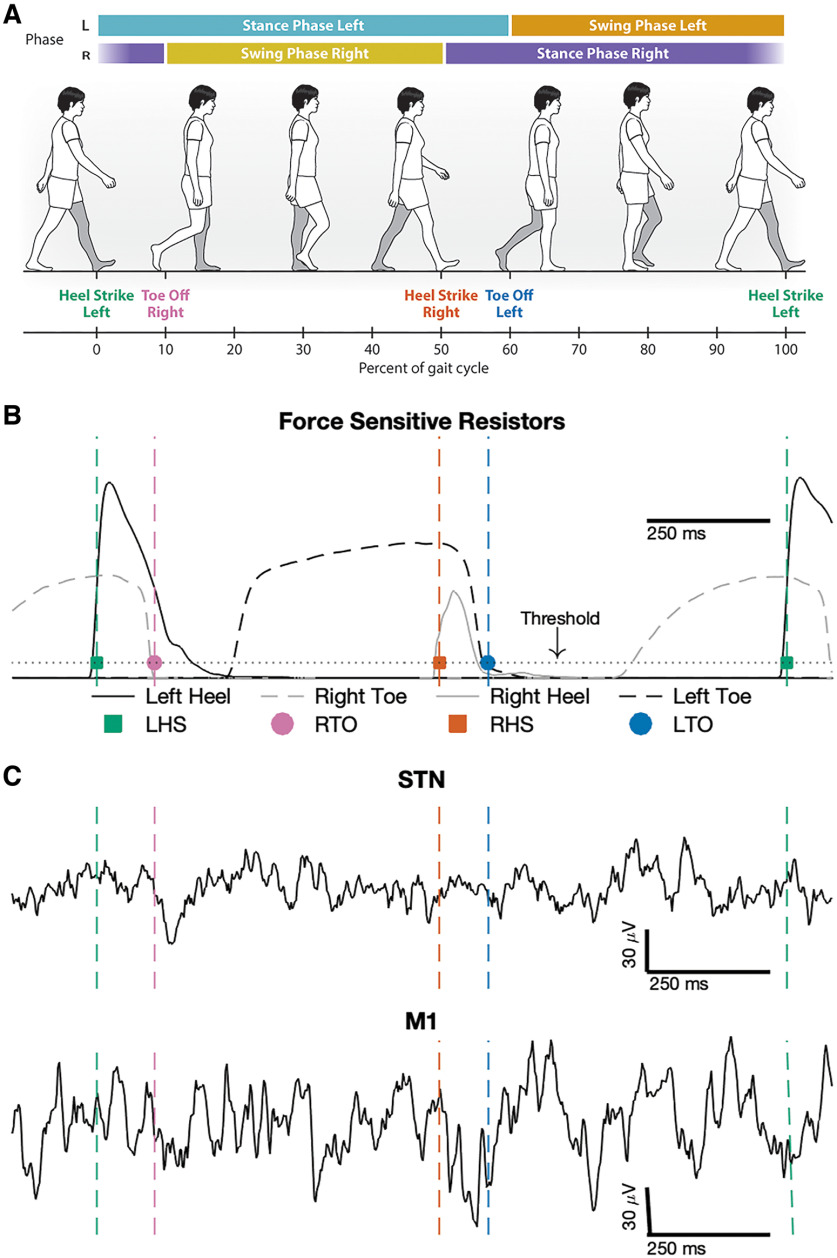
Synchronized gait kinematic data with raw local field potential recordings during natural walking. ***A***, Illustration of gait events and phases during a single gait cycle, aligned to left heel-strike (0% gait cycle). ***B***, Heel-strike (squares) and toe-off (circles) gait events were detected from the left (black) and right (gray) force-sensitive resistor data. Heel-strikes were detected when the heel force (solid line) exceeded a threshold (dotted line), and toe-offs were detected when toe force (dashed line) fell below the threshold. ***C***, Example local field potential recordings from both STN and M1 synchronized to a gait cycle. Left heel-strike (LHS), Right toe-off (RTO), Right heel-strike (RHS), Left toe-off (LTO).

Gait kinematic data were used to determine left and right toe-off and heel-strike events using a custom MATLAB script (Fig. 2). Heel-strike was defined as the time when the calcaneus or 5MT FSR crosses over a 5% threshold in the positive direction. Toe-off was defined as the time when the hallux or 1MT FSR crosses over the 5% threshold in the negative direction. For the Xsens system, toe-off was defined as the time of peak ankle plantarflexion velocity, while heel-strike was defined as the time of ankle velocity impulse. All gait events were visually inspected, and erroneous events were manually corrected. Turns were excluded from analysis. Forty gait cycles were included for analysis from subject 1, 67 for subject 2, and 106 for subject 3.

Individual gait cycle epochs were extracted from the CWT and wavelet coherence data and were divided into time bins representing 1% of the gait cycle. Power and magnitude-square coherence values for each gait cycle were normalized to the average value during the entire walking period by *z* score. The *z*-cored values for gait cycles were then averaged across subjects to obtain the grand average spectrogram and coherogram.

To identify frequency bands where power differed between gait events, instantaneous power at each gait event (left and right toe-off and heel-strike) were extracted. All possible frequency bands were created between 0 and 50 Hz, and a Kruskal–Wallis test was used to identify frequency bands where power differed among the gait events. A Kruskal–Wallis test was used because the datasets were not normally distributed (Shapiro–Wilk test), but the variances of the different gait events were equal (Levene’s test). The *p*-values were adjusted using Tukey’s honestly significant difference method. Frequency bands where the multiple-comparisons test reached *p*-values < 0.05 were designated as gait event-modulated frequency bands.

### Gait event classification

A classification model was built to predict gait events from LFP power and the STN–cortical coherence. The classification model used an ensemble learning approach to enhance the stability and accuracy (Wolpert, 1992; Polikar, 2006) and consisted of a random forest (RF) feature selection model and a linear discriminant analysis (LDA) model. RF has been shown to achieve better performance than other feature selection methods (Chen et al., 2020) and is robust to collinearity (Genuer et al., 2010). The LDA model matched the on-board hardware classifier of the RC+S. The classifier models were built in R with the “Tidymodel” framework (https://www.tidymodels.org/) and trained for each subject, brain hemisphere, and recording area.

Features used in the RF model were instantaneous power or magnitude-squared coherence during toe-off events in all possible frequency bands between 2.5 and 50 Hz. All features were normalized to a mean of 0 and an standard deviation of 1. Before feature selection, RF hyperparameters, the number of decision tress and the number of features a tree considers during node splitting, were optimized using 10-fold cross-validation with each dataset stratified by toe-off classes. Once optimized, the RF feature selection model was trained on all normalized features using the “ranger” (Wright and Ziegler, 2017) package in RStudio (www.rstudio.com).

The top 10 features with the largest variable importance value based on “permutation importance” (Altmann et al., 2010) were used to generate new datasets for each subject and brain hemisphere. Next, the new datasets were split into 75% for training and 25% for testing. The accuracy and receiver operator characteristic area under the curve (AUC) were calculated.

### Statistical analysis

A linear repeated-measures mixed model was used to determine power or coherence values that differed from the average during the gait cycle. A single fixed effect was used, and subjects were added to the model as a random to account for individual baseline neural power differences. Significance was tested using *F*-tests with the Satterthwaite degrees of freedom method. Statistical analysis of classification models was performed only on models that achieved greater than chance accuracy (≥50%). Significance was tested by permuting the toe-off class labels 1000 times and by calculating the class accuracy on the permuted data. Models were determined to be significant if it correctly classified the event in <5% of the total number of permutations (Herrojo Ruiz et al., 2014).

## Results

### STN shows coordinated low-frequency power modulation during walking

**Figure 3.**
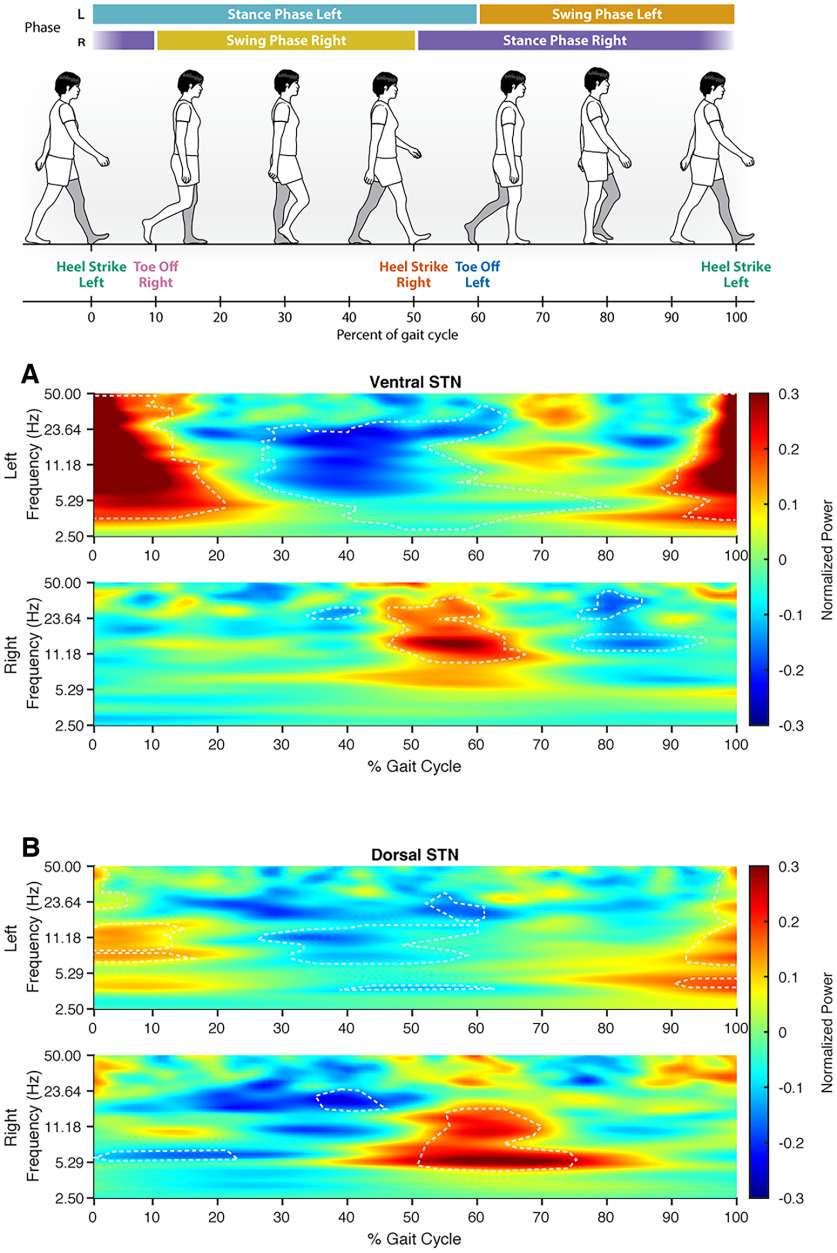
STN local field potentials show spectral power modulations during the gait cycle. Grand average *z* score spectrograms from the dorsal and ventral STNs normalized to a gait cycle. ***A***, ***B***, Significant power increases are seen during weight acceptance of the left leg in the left hemisphere (∼0–10% gait cycle) and right leg in the right hemisphere (∼50–60% gait cycle). Power increases were observed in a wide frequency band (10–50 Hz) in the ventral STN and in the low-frequency band (5–15 Hz) in the dorsal STN. Significant beta (13–30 Hz) desynchronization was also seen during contralateral leg swing and heel-strikes. ***A***, ***B***, Gait cycle percentages and frequencies where power was significantly different compared with the average power during the entire walking task is outlined by the dashed white lines. A linear mixed-effect model was used to determine significance with *p*-value < 0.05. (Extended Data Fig. 3-1 shows grand average gait cycles from cortical recorded contacts. Extended Data Fig. 3-2 shows a single gait cycle from all recorded areas from all subjects in the study and shows alternating left–right power changes throughout the gait cycle.)

To investigate STN and sensorimotor cortical neural dynamics during the gait cycle, we extracted and averaged spectral power across all gait cycle epochs and tested whether the power significantly changes during the gait cycle. We found that the two hemispheres showed coordinated and reciprocal changes in spectral power within the ventral and dorsal STN during the gait cycle. Significant changes in power were seen in the alpha to low-gamma frequency (10–50 Hz) band power in the ventral STN and in low-frequency (5–15 Hz) band power in the dorsal STN. Increased power occurred during the double support phase, the period from ipsilateral heel-strike to contralateral toe-off (0–10% for the left leg; 50–60% for the right leg; Fig. 3*A*,*B*, top). The left STN also demonstrated significant alpha-beta (8–30 Hz) power decrease during right leg swing period, and beta band (13–30 Hz) decrease during right heel-strike (Fig. 3*A*,*B*, top). These changes in LFP power were also seen in individual gait cycles across all subjects (Extended Data Fig. 3-2*A*,*B*).

10.1523/ENEURO.0325-22.2022.f3-1 Figure 3-1 Cortical local field potentials show spectral power modulations during the gait cycle. ***A***, Left, M1 shows alpha (8–10 Hz) and beta desynchronization during right leg heel strike and initial right leg swing, respectively. Right, M1 shows increased theta–alpha (5–12 Hz) synchronization during initial left leg swing and decreased beta around left heel-strike. ***B***, Significantly decreased beta power is seen during left leg weight acceptance and initial right leg swing. Increases in theta-beta power (5–23 Hz) were seen during weight acceptance of the right leg and initial left leg swing. Download Figure 3-1, TIF file.

10.1523/ENEURO.0325-22.2022.f3-2 Figure 3-2 Individual gait cycle spectrograms. Spectrograms of a single gait cycle from the STN and sensorimotor cortices. All subjects show alternating left and right spectral power changes throughout the gait cycle. Download Figure 3-2, TIF file.

M1 and S1 also demonstrated power changes throughout the gait cycle, though the frequency-specific changes between the left and right hemispheres were not reciprocal. The left M1 showed decreased beta activity during right leg swing (10–30% of gait cycle) and increased beta power during right leg stance (60–80% of gait cycle; Extended Data Fig. 3-1*A*, top). While the right M1 does not show significant beta power modulation, it showed theta power changes during the end of right leg swing and the beginning of left leg swing (Extended Data Fig. 3-1*A*, bottom). The right S1 shows a similar pattern of theta modulation during transition from right leg swing to left leg swing (Extended Data Fig. 3-1*B*, bottom).

### STN interacts with motor and sensory cortices during different phases of the gait cycle

**Figure 4.**
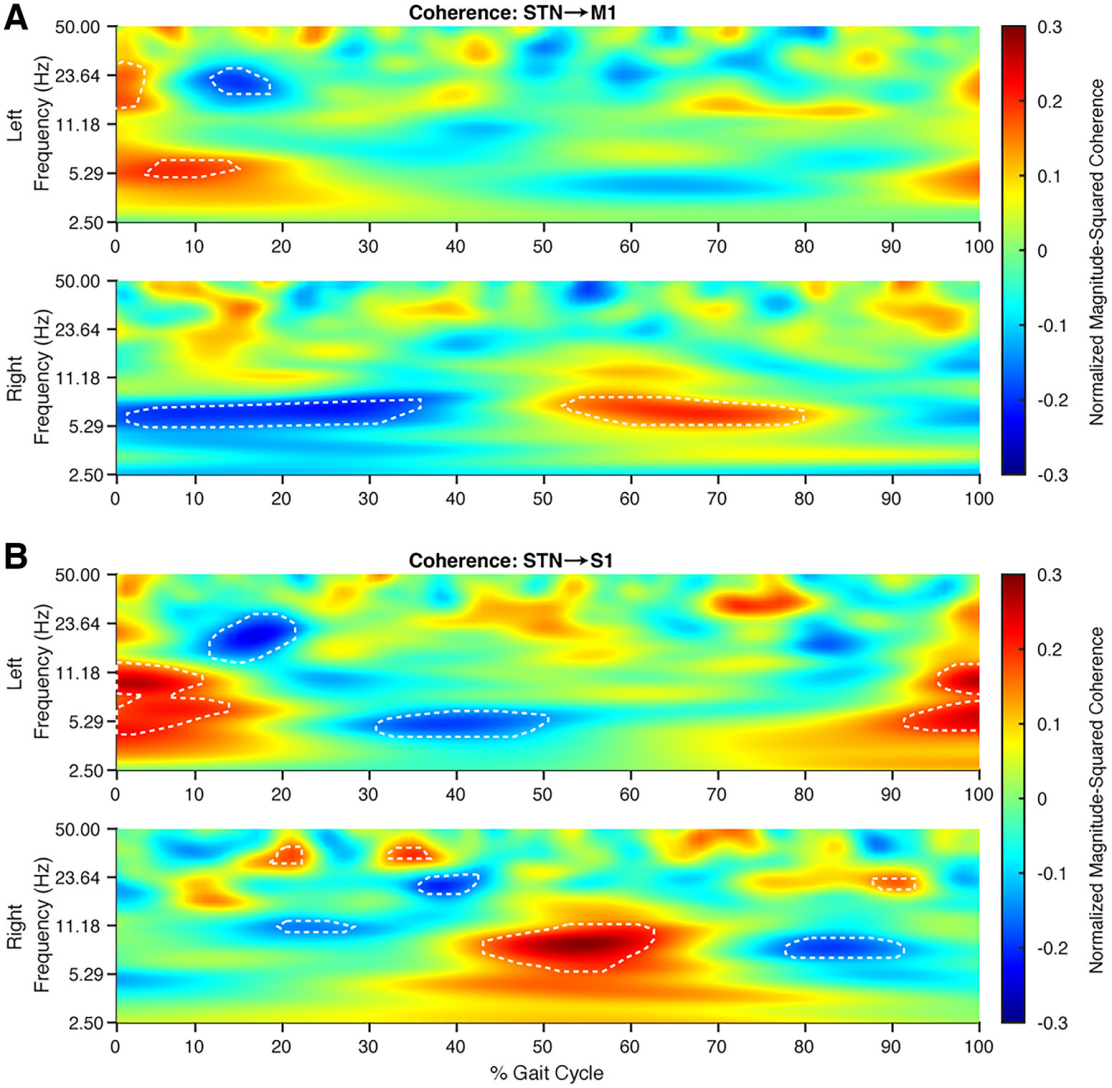
Low-frequency STN/cortical coherence increase during the initiation of contralateral leg swing. Grand average *z* score coherogram from STN–M1 and STN–S1 normalized to a gait cycle. Reciprocal coherence modulation was seen in both hemispheres. ***A***, STN–M1 coherence showed significant increases in the theta band (5–8 Hz) during the initiation of contralateral leg swing through mid-swing. Additionally, the left hemisphere showed beta band coherence increases during initial ipsilateral weight acceptance. ***B***, STN–S1 coherence modulation was seen theta/alpha band across both hemispheres during ipsilateral heel-strike. ***A***, ***B***, Gait cycle percentages and frequencies where coherence was significantly different from the average coherence during the entire walking task are outlined by the dashed white lines. A linear mixed-effect model was used to determine significance with a *p*-value < 0.05.

Because the STN has direct connections with sensorimotor cortices and plays important functions in motor control, we examined whether the STN interacts with the cortex during specific phases of the gait cycle. To determine the nature and degree of this interaction, we compared the averaged magnitude-squared coherence value between the STN and M1/S1 for each brain hemisphere during the gait cycle. We found increased STN–M1 theta band coherence during contralateral toe-off and initial contralateral leg swing, similar to the power modulations seen in the STN (Fig. 4*A*). Interesting, STN–S1 showed greater theta and alpha band coherence during ipsilateral heel-strike (Fig. 4*B*). The two brain hemispheres showed reciprocal coherence modulations.

### Patient-specific oscillatory biomarkers of gait

**Figure 5.**
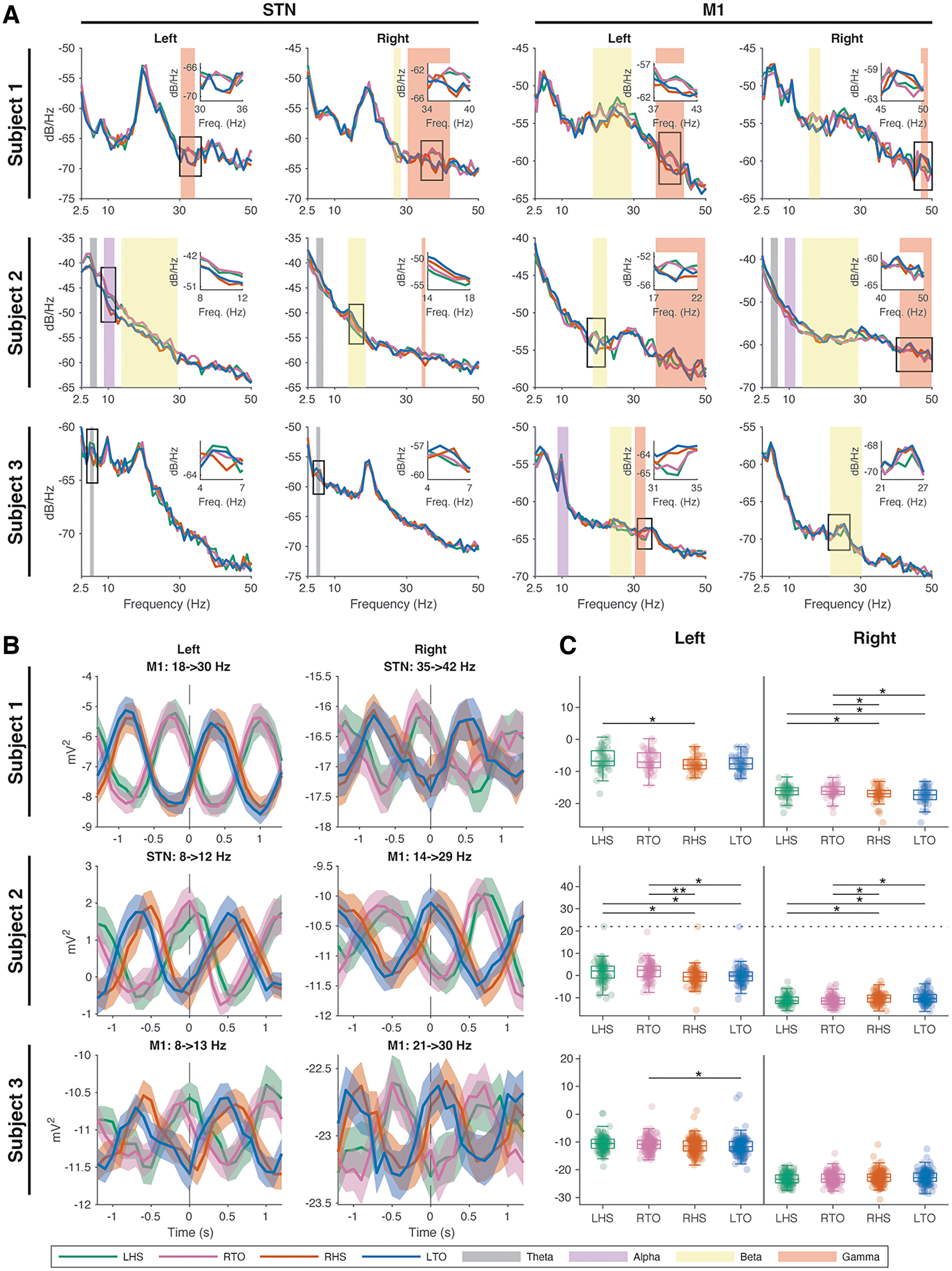
Unique frequency bands within each subject can differentiate gait events. ***A***, Average heel-strike and toe-off PSDs from the STN and M1. Each subject had unique frequency bands where power during heel-strikes (green, left heel-strike (LHS); orange right heel-strike (RHS)) and toe-off (blue, left toe-off (LTO), pink right toe-off (RTO)) gait events were significantly different (*p* < 0.05). The unique frequency bands were mainly found within the canonical frequency ranges (color of shaded area), but rarely spanned the entire range (width of shaded area). Inset plots show power differences between gait events temporally distinct from each other in relation to the gait cycle. ***B***, Average power and standard error ± 1 s around the gait event. Reciprocal power modulation, offset by half a gait cycle, is seen between temporally distinct gait events in all subjects. Furthermore, all left hemisphere data show higher power during left heel-strike/right toe-off, and most of the right hemisphere data show higher power during right heel-strike/left toe-off. ***C***, Boxplot of gait event power within the frequency bands from ***B***. Individual gait event powers are shown as transparent colored dots with outliers shown on the dotted line. Multiple-comparison tests were performed against each pair of gait event within the same hemisphere. Level of significance is indicated as follows: *p < 0.05, **p < 0.005. (Extended Data Fig. 5-1 shows a visualization of the arbitrary length frequency bands created an Kruskal-Wallis *p*-value heat-map.)

Because our data showed several distinct gait-related frequency bands of modulation during the gait cycle, we used a data-driven approach to determine patient-specific frequency bands that are putative biomarkers for heel-strike and toe-off events. We created frequency bands of varying lengths ranging from 0 to 50 Hz, extracted power spectral density values at each gait event, and performed an Kruskal-Wallis for each band (Extended Data Fig. 5-1). We found that each patient had unique frequency bands where power values differentiated gait events (Fig. 5). Significant gait event-modulated frequency bands were found within all canonical frequency bands, with a majority in the theta and beta bands (Fig. 5*A*). Frequency ranges of the gait event-modulated bands varied by electrode location but were typically a subrange of the canonical bands. By comparing the instantaneous power spectral density during each of the four gait events, we found power differences between gait events that are temporally distinct (Fig. 5*A*, inset plots), whereas gait events occurring in temporal proximity have a more similar power spectra profile (Fig. 5*A*).

10.1523/ENEURO.0325-22.2022.f5-1 Figure 5-1 Example arbitrary frequency bands and Kruskal–Wallis testing (related to Fig. 5). Varying lengths of frequency bands were created between 0 and 50 Hz. Each frequency is referenced as a bin. Start and end bins refer to the varying lengths of frequency band for start and end frequencies. Power during left and right heel-strike and toe-off events were extracted from each frequency band and an Kruskal–Wallis test was performed. The *p*-value of the Kruskal–Wallis test was stored, and a heat map was created. Example of the resulting heat map is shown from subject 2 M1 recorded area. Significant Kruskal–Wallis test outcomes can be observed to fall within the low-gamma band (35–45 Hz) frequency. Download Figure 5-1, TIF file.

To evaluate how the amplitudes of these gait-specific biomarkers change over the gait cycle, we the averaged their power over a 1 s period around each gait event and found them to fluctuate for the duration of the gait cycle (Fig. 5*B*). Power averages for the left heel-strike and right toe-off events are offset by half a gait cycle to the right heel-strike and left toe-off events. In all subjects, the left and right hemispheres showed reciprocal power modulations across different contacts.

To investigate whether the instantaneous powers of each gait event are distinct from each other, a multiple-comparisons test was performed between all possible pairs of gait events. Significant power differences were found between left and right heel-strikes in subjects 1 and 2 in both hemispheres (Fig. 5*C*). Other significant differences occurred between toe-off events (Fig. 5*C*). Gait events temporally close to each other did not differ in power.

### Decoding gait events based on cortical and subcortical LFPs

**Figure 6.**
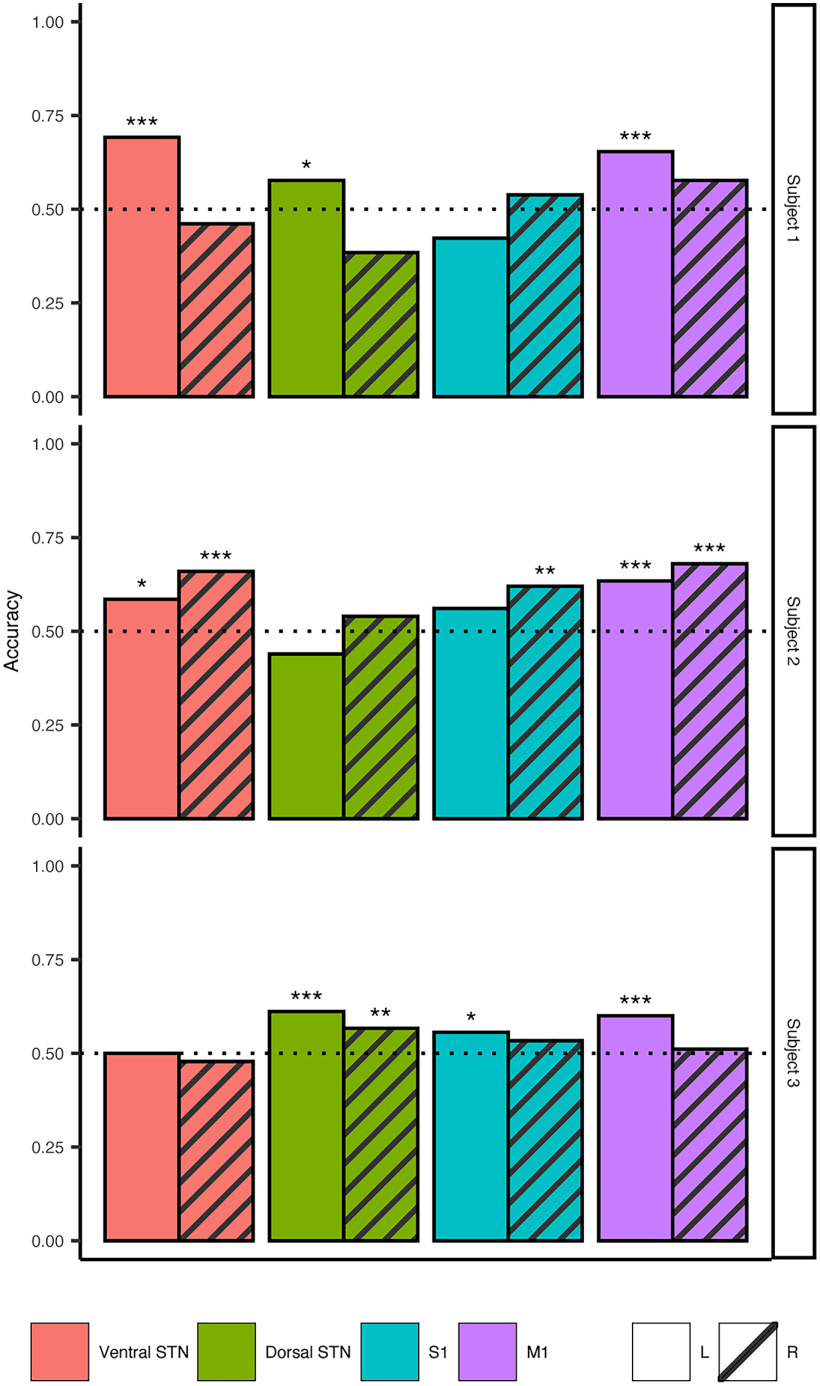
Gait event decoding using oscillatory features achieves greater than chance accuracy. LDA ensemble classifiers were trained on left and right toe-off events for each contact and hemisphere across all subjects. All subjects had at least one contact where the classification accuracy of at least one model was ≥61.1%. Maximum accuracy achieved across all subjects were between 61.1% and 69.2%. Maximum discriminatory ability was calculated using the area under the receiver operator characteristic curve and ranged between 0.585 and 0.763. Each subject’s models are shown on each row. The recorded area the LDA model was built from is indicated in color and follows this order (left to right): red, ventral STN; green, dorsal STN; blue, S1; purple, M1. The bar pattern indicates the brain hemisphere the model was built from: solid, left hemisphere; striped, right hemisphere. *p < 0.05, **p < 0.005, ***p < 0.0005. (Extended Data Fig. 6-1 shows results from classifier models built using coherence values between the STN and M1.)

Based on finding spectral signatures for specific gait events of the gait cycle, we wanted to decode gait events using these personalized “gait biomarkers.” Using the LDA model, we were able to classify toe-off events with ≥61% accuracy (Table 2) in all subjects from at least one of the recorded contacts (Fig. 6). Significant above-chance accuracy was achieved from models built using left and right hemisphere data in subjects 2 and 3, but only from left hemisphere-trained models in subject 1. No electrode location outperformed others consistently but was subject specific. Overall, the median model accuracies were greater than chance and ranged from 54.4% to 60.3%. Further analysis of the models showed the maximum discriminatory value achieved, evaluated by calculating the AUC, ranged between 0.585 and 0.763.

**Table 2:** Classification summary

ID	Median accuracy	Maximum accuracy	Median AUC	Maximum AUC
Subject 1	55.8%	69.2%	0.592	0.763
Subject 2	60.3%	68.0%	0.635	0.733
Subject 3	54.4%	61.1%	0.574	0.585

10.1523/ENEURO.0325-22.2022.f6-1 Figure 6-1 Toe-off gait event decoding using STN–M1 coherence. LDA ensemble classifiers were trained using coherence magnitude squared values between the ventral and dorsal STN to M1 and S1. The highest accuracy and discriminatory values achieved were similar to models built from individual recorded areas. The highest accuracy values achieved were between 58.9% and 68.3%, and the highest discriminatory values were between 0.602 and 0.786. Each subject’s models are shown in each row. The recorded area the LDA model was built from is indicated in color and follows this order (left to right): pink, ventral STN; yellow, dorsal STN; brown, S1; orange, M1. Bar patterns indicate the brain hemisphere the model was built from: solid, left hemisphere; striped, right hemisphere. *p < 0.05, **p < 0.005, ***p < 0.0005. Download Figure 6-1, TIF file.

We also explored whether coherence between STN and M1/S1 could classify toe-off events. The subcortical–cortical coherence pair that achieved the highest accuracy was subject specific, and only subjects 2 and 3 had models reach significant above-chance accuracy (accuracy, 58.9–68.3%; AUC, 0.602–0.786; Extended Data Fig. 6-1).

## Discussion

We used chronic invasive recordings in PD patients to advance our understanding of dynamic subthalamic and sensorimotor oscillatory changes that underlie natural overground walking. First, we demonstrate the novel finding that STN displays increased low-frequency (4–12 Hz) activity during the double support period before contralateral leg swing. Furthermore, STN shows increased theta frequency coherence with the primary motor during initiation of contralateral leg swing, implicating a potential mechanism for the supraspinal network to scale and fine-tune leg muscle activation during stepping. Our findings support the hypothesis that oscillations from the basal ganglia and cortex direct alternating power fluctuations between the two hemispheres, in that power is offset by half a gait cycle, which may indicate a mechanism to coordinate and maintain continuous bipedal locomotion in humans. In addition, we identified patient-specific, gait-related biomarkers in both subcortical and cortical areas at discrete frequency bands. Exploratory ensemble classification models showed above-chance accuracy in classifying left and right gait events using oscillatory power features.

### Alternating multifrequency modulations from bilateral STNs during gait

Several groups have described beta power modulations within the STN during the gait cycle between the left and right hemispheres during seated stepping (Fischer et al., 2018) and overground walking (Arnulfo et al., 2018; Hell et al., 2018; Canessa et al., 2020) in Parkinson’s disease patients. Because elevated beta synchrony within the STN is associated with the akinetic state in Parkinson’s disease, it is logical that beta desynchronization is required for movement, including gait. We found that these gait-event related alternating power modulations between the left and right STNs are not limited to the high beta-frequency range but also involve other low-frequency bands.

What are the roles of subthalamic lower frequency (theta and alpha) modulation during gait? Previous studies on upper extremity movement tasks have shown event-related theta/alpha frequency synchronization within the STN at the onset and throughout the duration of a sustained voluntary muscle contraction task (Tan et al., 2013; Kato et al., 2016). In some cases, the amplitude of these theta/alpha oscillations correlate with the force generated during hand movement (Anzak et al., 2012). STN theta activity has also been shown to have a role in the cognitive control of movement, such as during sensorimotor conflict (Aron et al., 2016; Zavala et al., 2017) and response inhibition (Alegre et al., 2013). We posit that these low-frequency oscillations emerge from the STN during periods of gait that require greater cortical engagement. Based on increases in STN theta/alpha power we found during the transition from double support (both feet on the ground) to single support (ipsilateral leg on the ground) period, we postulate that these low-frequency modulations engage multiple motor cortical areas to generate the appropriate scale and force required during contralateral leg swing to maintain stable single limb support and bipedal locomotion.

While some suggest that low-frequency modulations during gait may be secondary to movement-related artifacts (Hell et al., 2018), we believe that these low-frequency oscillations reflect physiological signals for several reasons. First, spectral activities that change during the gait cycle are focal in frequency range and are not broadband in nature (Fig. 3). Second, the spectral power changes in left and right STNs are offset by half a gait cycle, unlike in a previous study where both STNs showed concurrent spectral power increases during the gait cycle regardless of laterality (Hell et al., 2018). Finally, the dorsal and ventral STN, as well as M1 and S1 contacts connected to the same RC+S, show different time–frequency changes from each other during the gait cycle and, hence, are less likely to reflect artifacts.

One key question is whether these gait-related oscillatory modulations reflect physiological or pathologic gait patterns. While our patients did not have overt gait abnormalities such as shuffling gait or freezing of gait, they performed the walking task on dopaminergic medication, which can affect oscillatory activity (Foffani et al., 2006; Ray et al., 2008). Pallidal LFP recorded from patients with segmental dystonia without gait disorders has shown power modulations in the theta, alpha, and beta frequencies during gait (Singh et al., 2011), and demonstrated similar theta/alpha frequency power modulations during early stance and swing phase of the contralateral leg. While we cannot rule out the presence of compensatory signals in the disease state, we speculate that our results are an indicator of physiological gait, rather than pathologic. The dynamic changes of oscillations across different frequency bands may provide a mechanism to coordinate and recruit different cortical and subcortical areas in response to changes in posture, balance, and forward momentum during walking.

### Cortical–subthalamic interactions during gait

In a study involving simultaneous recording of STN LFPs and scalp EEG during walking in Parkinson’s disease patients with freezing of gait, the authors found elevated cortical–STN synchrony of 4–13 Hz during effective gait (Pozzi et al., 2019). The spatiotemporal specificity of field potentials captured by the permanently implanted cortical electrodes indicate distinct interactions between the STN and different cortical areas during gait. We demonstrated increased STN–S1 coherence in the low-frequency ranges (theta–alpha) during the double-support period between ipsilateral heel-strike and contralateral toe-off. We also found increased STN–M1 theta frequency coherence during contralateral toe-off and early contralateral leg swing. These alternations in coherence are offset by half a gait cycle between the left and right hemispheres. To our knowledge, this is the first report of distinct patterns of STN–S1 and STN–M1 synchrony during human gait. We speculate that increased STN–S1 coherence during ipsilateral heel-strike to contralateral toe-off may represent sensory integration during the double support period as one prepares for leg swing. Increases in STN–M1 theta coherence then follows during the initiation of contralateral leg swing, which may allow the motor cortex to regulate the force of leg muscle activation required to drive forward stepping during gait. While these M1–STN interactions may represent normal recruitment of leg muscles during weight acceptance and transfer phase of the gait cycle, they may also represent compensatory mechanisms by which greater cortical activity is required to drive and maintain locomotion in Parkinson’s disease.

### Gait event decoding and potential clinical significance

A key finding from our study was that, for each patient, a unique range of frequencies was significantly differentially modulated corresponding to the various gait events. While these frequency bands often overlap canonical bands, they are usually narrower and span many different canonical frequencies. The variations among patients may be because of slight differences in electrode placement. While our results show greater than chance median accuracy and acceptable to medium discriminatory ability, the models may be underoptimized for each subject. By constraining the set of possible hyperparameter values, possible values that would result in better accuracy and discriminatory ability for different subjects may have been missed. Additionally, the ratio of features (1770 total) compared with observations during feature selection can overfit the model, leading to poor feature selection. Nonetheless, our study demonstrates the feasibility of distinguishing gait events based on cortical or STN LFP power.

One of the reasons to identify gait-specific biomarkers is to use them as control signals for closed-loop, also known as adaptive DBS (aDBS). The Summit RC+S system implanted in our subjects allows for aDBS in real time and uses LDA to detect different brain states using Fourier transform power within a frequency band (Sellers et al., 2021; Ansó et al., 2022). The aDBS feature of the Summit RC+S device has been successfully tested in PD patients (Gilron et al., 2021a, b) and a cervical dystonia patient (Johnson et al., 2021), with a varying timescale for stimulation changes (from hundreds of milliseconds to minutes). Therefore, it is feasible to implement real-time aDBS to rapidly change stimulation parameters to improve gait function in Parkinson’s disease patients.

### Limitations

Our sample size is small because of invasive nature of these studies with investigational devices. Patients performed all tasks while receiving medication, which may affect beta power modulation. Because of variations in patient anatomy and electrode placement, M1 and S1 electrodes may capture different parts of the homunculus. Our event-related power modulation from the cortex may be related to arm movement rather than leg movement. However, in another study, we have observed that the motor cortex is attuned to different limb movements in different frequency ranges (i.e., greater beta modulation during arm swing vs greater theta modulation during leg movement; C.K. Starkweather, M.A. Morrison, M.S. Yaroshinsky, K.H. Louie, J. Balakid, K. Presbrey, P.A. Starr, D.D. Wang, unpublished data). Additionally, there is increasing evidence pointing to the existence of intermixed neural tuning of the whole body, including leg and foot movement, in the “hand knob” area of the precentral gyrus in humans (Zeharia et al., 2012; Willett et al., 2020).

### Conclusion

This study provides new insights on the role that subthalamic and sensorimotor oscillations play in human gait. Our data also support the notion that the STN and sensorimotor cortices contain patient-specific, gait-related frequency modulations that can be used to distinguish between left and right gait events. This knowledge has the potential to be integrated into adaptive neuromodulation therapies to improve gait functions in patients with Parkinson’s disease.

## Synthesis

Reviewing Editor: Laura Volpicelli-Daley, The University of Alabama at Birmingham Decisions are customarily a result of the Reviewing Editor and the peer reviewers coming together and discussing their recommendations until a consensus is reached. When revisions are invited, a fact-based synthesis statement explaining their decision and outlining what is needed to prepare a revision will be listed below. The following reviewer(s) agreed to reveal their identity: Jeevan Kumar Jadapalli. Note: If this manuscript was transferred from JNeurosci and a decision was made to accept the manuscript without peer review, a brief statement to this effect will instead be what is listed below.

Both reviewers feel the study is an interesting and novel approach to addressing connectivity between STN and cortex and gait. Reviewer 1 felt the concerns about statistics have not been addressed and that a one-way ANOVA is not justified. Please consult with a biostatistican and return the manuscript with new statisical analyses. Also the review asked for some clarifications in methods and figure legends.

This is an interesting novel approach, that attempts to address the local field potential connectivity between STN and Cortex during the gait cycle, even though the limitations of this research were clearly stated, a few additional comments need to be addressed to improve clarification

Line 87: In methods, when the lead localization is done is not clear? It says post-operative but most likely leads placement will be altered if the localization is not verified after the surgery recovery.

Line 102: are these DBS contact locations are same for all 3 patients, or the mentioned text is for one individual is not clear in the text?

Line 268: Please re-do the stats or contact a biostatistician for assistance, even though the authors try to justify for one-way ANOVA- the presented data doesn’t meet the requirements of ANOVA (sample size, normality, variance, and homoscedasticity)

Figure 3 and 4: Both figure legends state grand averaged spectrograms normalized to gait cycle, are these grand averaged across time duration 15 sec in single participant, or is the plot is average of all 3 participants unclear? Event-related spectral perturbation method will be the more appropriate method. if the authors don’t want to lose any phasal data since averaging two wave signals, leads to a change in phase.

Figure 5B: appropriate reference is needed for the proposed method in finding phase/ synchrony. Besides, there are more robust techniques to find phase relationships such as phase-amplitude coupling, which gives a more accurate robust measure in identifying if there is any phasal/synchronal relationship of STN signals with cortical signals.

Minor comment:

Figure 3: it will be interesting to compare dorsal and ventral STN, but since the color bars are on different scales, it gives the reader the wrong interpretation.

Print Report

## Author Response

Line 87: In methods, when the lead localization is done is not clear? It says post-operative but most likely leads placement will be altered if the localization is not verified after the surgery recovery.

We have edited the text to include what images were used to create the DBS and cortical electrode localization.

"DBS and cortical electrode localization were performed using 2-month postoperative CT images fused with preoperative T1-weighted MRI images. STN DBS lead reconstruction...”

Line 102: are these DBS contact locations are same for all 3 patients, or the mentioned text is for one individual is not clear in the text?

We have changed the text to clarify that these recording contacts were used across all patients.

"In all subjects LFPs were recorded from two STN electrode pairs: ventral STN (+2-0) and dorsal STN (+3-1), where contact 0 is in the ventral STN, contact 3 just above the dorsal border, and contacts 1 and 2 in the motor territory based on microelectrode mapping (Fig. 1A).”

Line 268: Please re-do the stats or contact a biostatistician for assistance, even though the authors try to justify for one-way ANOVA- the presented data doesn’t meet the requirements of ANOVA (sample size, normality, variance, and homoscedasticity)

We appreciate the suggestion from the reviewer and to address the concern, we have changed the analysis. We first checked the normality of our data set using the Shapiro-Wilks test, which told us our data is not normally distributed. Next, we tested whether the variances of the nominal variable (i.e. the four gait events) were equal using Levene’s test. The result of Levene’s test indicated that the variances of our nominal variable were equal. Thus, instead of an ANOVA test, we performed a Kruskal-Wallis test. We have updated the text in the manuscript (Lines 145-149) and is included below. We have removed Table 2 as it is no longer relevant. We have updated the figure and is included below.

"All possible frequency bands were created between 0-50 Hz, and a Kruskal-Wallis test was used to identify frequency bands where power differed among the gait events. A Kruskal-Wallis test was used because the data sets were not normally distributed (Shapiro-Wilk test), but the variances of the different gait events were equal (Levene’s test).”

Figure 3 and 4: Both figure legends state grand averaged spectrograms normalized to gait cycle, are these grand averaged across time duration 15 sec in single participant, or is the plot is average of all 3 participants unclear? Event-related spectral perturbation method will be the more appropriate method. if the authors don’t want to lose any phasal data since averaging two wave signals, leads to a change in phase.

We appreciate the suggestion by the reviewer and would like to clarify the experimental protocol and analysis. The participants walked continuously for 2 minutes in a loop with a straight path of at least 15 feet, turns are excluded from all analysis. A single gait cycle is defined as time between two consecutive left heel strikes. Figures 3 and 4 are average gait cycle spectrogram plots of the three participants together. Each participant’s gait cycles were z-scored normalized to the entire walking period before averaging across participants. Normalizing to the entire walking period was chosen because we expect a baseline change in neural activity while the participants are upright and walking, compared to quiet stance.

To perform the suggested event-related spectral perturbation (ERSP) analysis (Makeig, 1993), we would need individual epochs of a single gait cycle and a baseline period prior to the first gait event. Given that the participants walked continuously, we do not have a standalone epoch of each gait cycle. Therefore, the ERSP analysis would not be appropriate for our data set.

Figure 5B: appropriate reference is needed for the proposed method in finding phase/ synchrony. Besides, there are more robust techniques to find phase relationships such as phase-amplitude coupling, which gives a more accurate robust measure in identifying if there is any phasal/synchronal relationship of STN signals with cortical signals.

We should clarify that we are not examining the phase relationship of the LFP signals but analyzing the power fluctuations at various phases of the gait cycle (i.e. left and right heel strike and toe-off). We will clarify this in our text. We did examine phase locking of the gait-related frequency bands at various gait events and did not see any relationship (plot below). The plot is a polar histogram of the alpha band frequency phase.

Minor comment:

Figure 3: it will be interesting to compare dorsal and ventral STN, but since the color bars are on different scales, it gives the reader the wrong interpretation.

We have changed the color scale to match between dorsal and ventral STN spectrogram plots.
