# Supplementary figures and images for: Cortico-Subthalamic Field Potentials Support Classification of the Natural Gait Cycle in Parkinson’s Disease and Reveal Individualized Spectral Signatures
Source: eNeuro. 2022 Nov 10;9(6):ENEURO.0325-22.2022. doi: 10.1523/ENEURO.0325-22.2022 (PMC9663205; doi:10.1523/ENEURO.0325-22.2022)

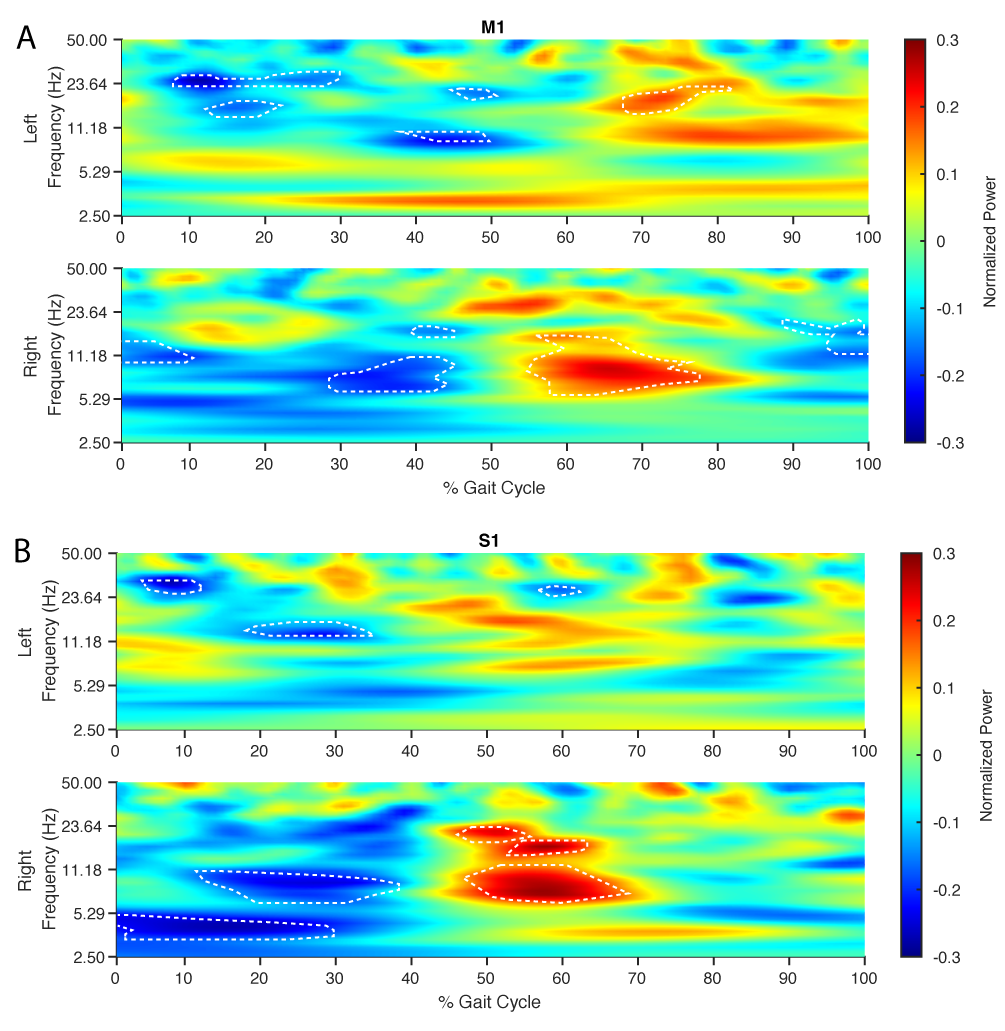

Supplement: Figure 3-1 — Cortical local field potentials show spectral power modulations during the gait cycle. A, Left, M1 shows alpha (8–10 Hz) and beta desynchronization during right leg heel strike and initial right leg swing, respectively. Right, M1 shows increased theta–alpha (5–12 Hz) synchronization during initial left leg swing and decreased beta around left heel-strike. B, Significantly decreased beta power is seen during left leg weight acceptance and initial right leg swing. Increases in theta-beta power (5–23 Hz) were seen during weight acceptance of the right leg and initial left leg swing. Download Figure 3-1, TIF file. [file enu-eN-NWR-0325-22-s02.tif]

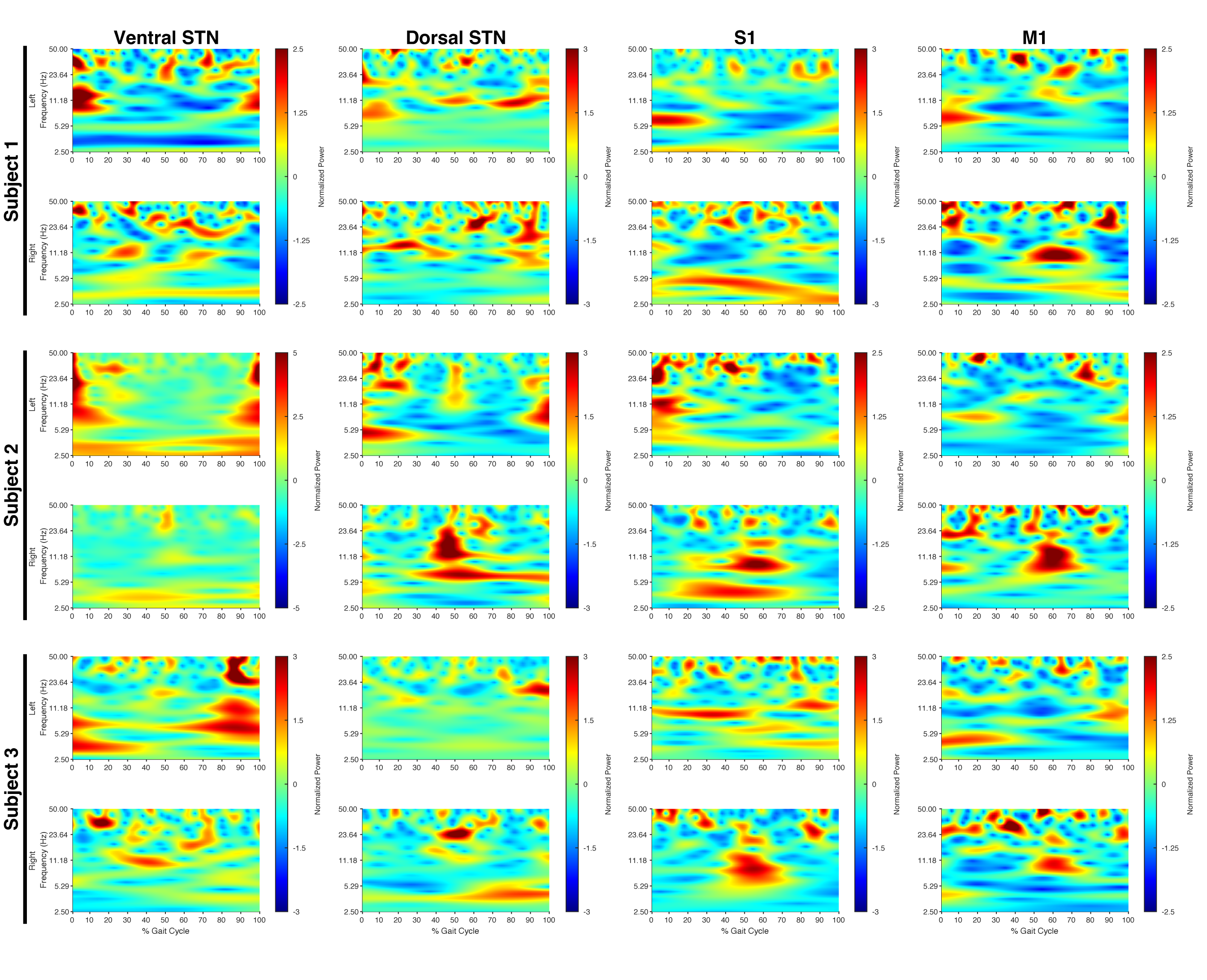

Supplement: Figure 3-2 — Individual gait cycle spectrograms. Spectrograms of a single gait cycle from the STN and sensorimotor cortices. All subjects show alternating left and right spectral power changes throughout the gait cycle. Download Figure 3-2, TIF file. [file enu-eN-NWR-0325-22-s03.tif]

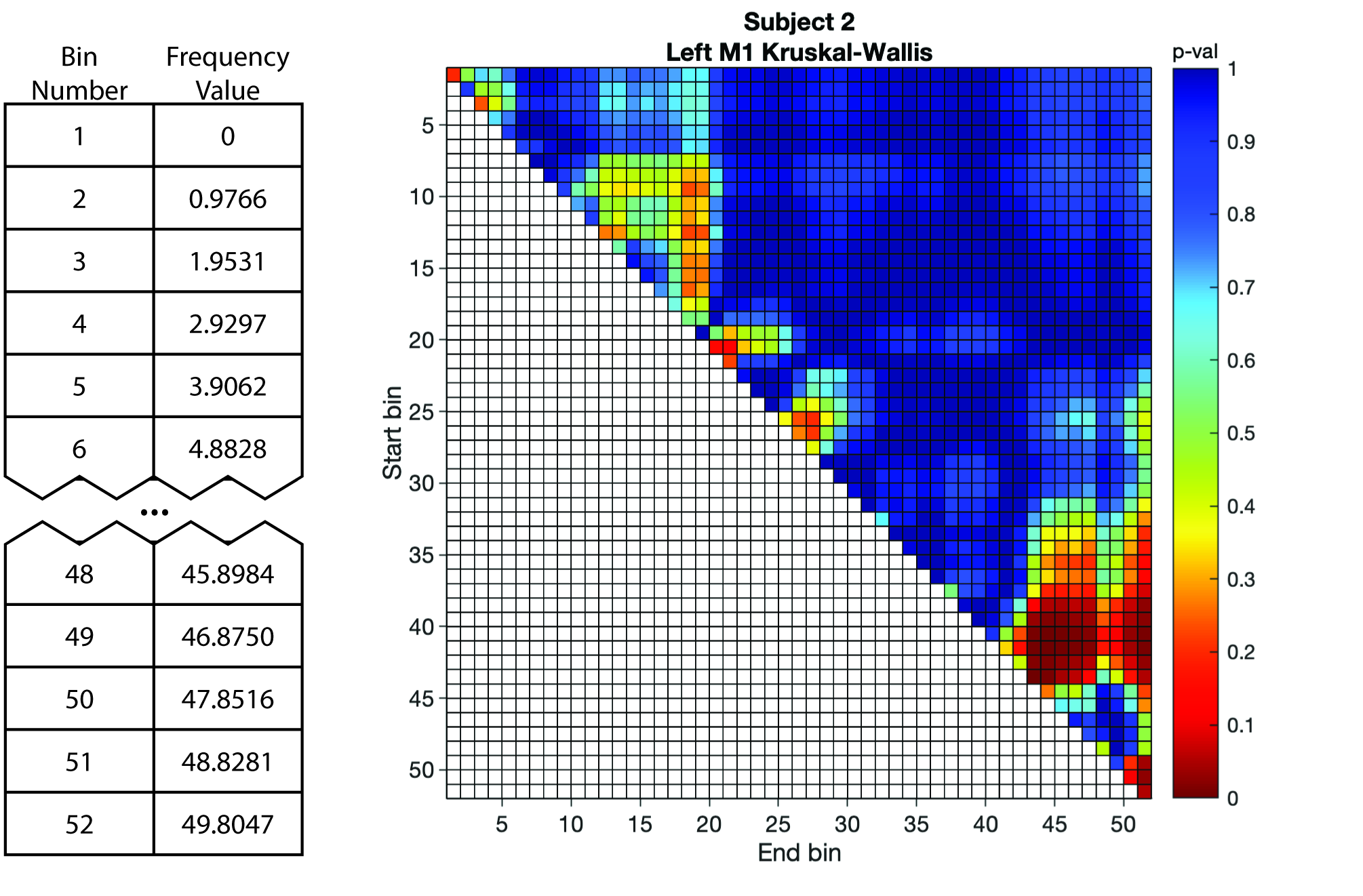

Supplement: Figure 5-1 — Example arbitrary frequency bands and Kruskal–Wallis testing (related to Fig. 5). Varying lengths of frequency bands were created between 0 and 50 Hz. Each frequency is referenced as a bin. Start and end bins refer to the varying lengths of frequency band for start and end frequencies. Power during left and right heel-strike and toe-off events were extracted from each frequency band and an Kruskal–Wallis test was performed. The p-value of the Kruskal–Wallis test was stored, and a heat map was created. Example of the resulting heat map is shown from subject 2 M1 recorded area. Significant Kruskal–Wallis test outcomes can be observed to fall within the low-gamma band (35–45 Hz) frequency. Download Figure 5-1, TIF file. [file enu-eN-NWR-0325-22-s04.tif]

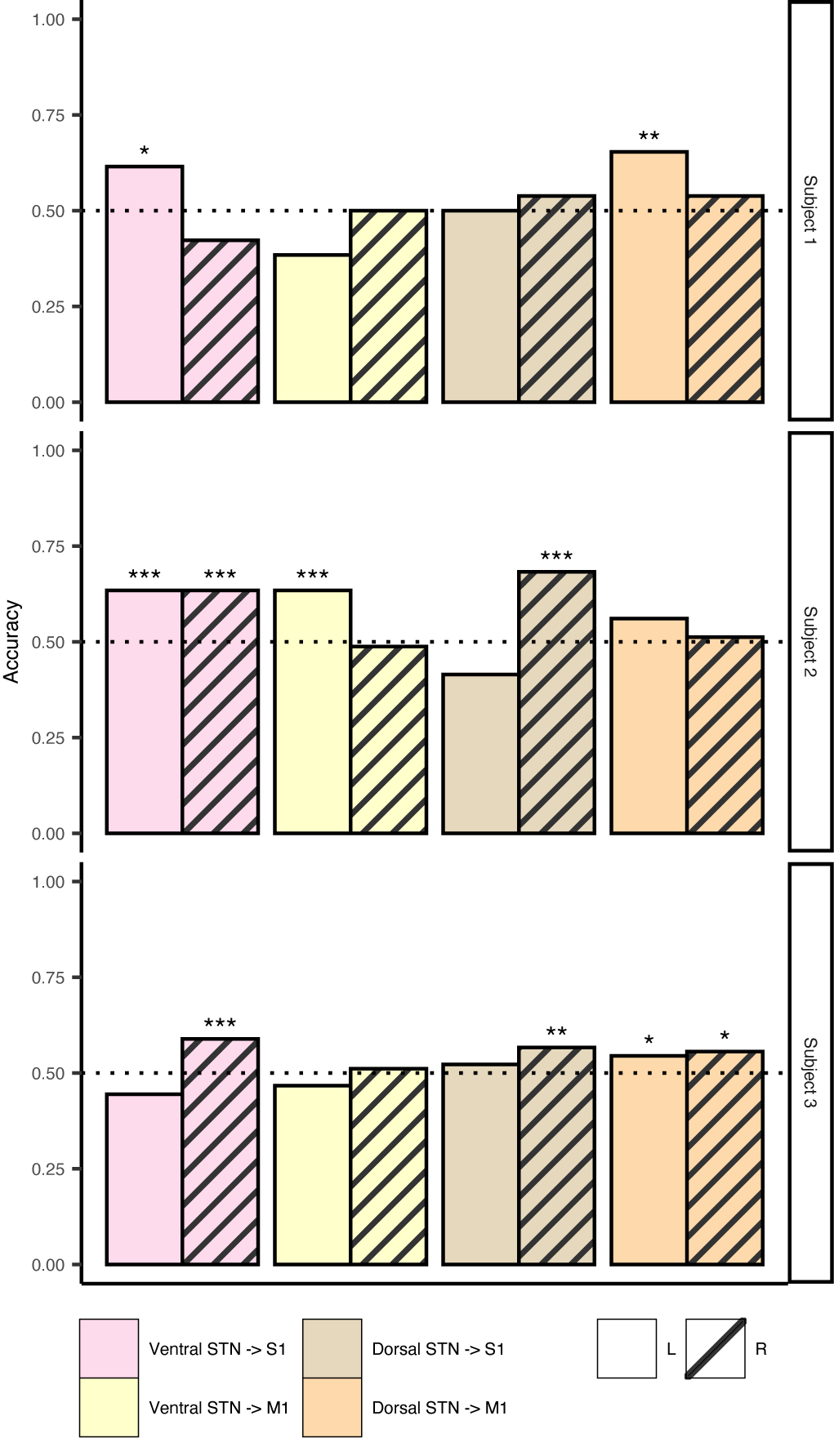

Supplement: Figure 6-1 — Toe-off gait event decoding using STN–M1 coherence. LDA ensemble classifiers were trained using coherence magnitude squared values between the ventral and dorsal STN to M1 and S1. The highest accuracy and discriminatory values achieved were similar to models built from individual recorded areas. The highest accuracy values achieved were between 58.9% and 68.3%, and the highest discriminatory values were between 0.602 and 0.786. Each subject’s models are shown in each row. The recorded area the LDA model was built from is indicated in color and follows this order (left to right): pink, ventral STN; yellow, dorsal STN; brown, S1; orange, M1. Bar patterns indicate the brain hemisphere the model was built from: solid, left hemisphere; striped, right hemisphere. *p < 0.05, **p < 0.005, ***p < 0.0005. Download Figure 6-1, TIF file. [file enu-eN-NWR-0325-22-s05.tif]
